# THP-1 Cells and Pro-Inflammatory Cytokine Production: An In Vitro Tool for Functional Characterization of NOD1/NOD2 Antagonists

**DOI:** 10.3390/ijms20174265

**Published:** 2019-08-30

**Authors:** Žiga Jakopin, Emanuela Corsini

**Affiliations:** 1 Faculty of Pharmacy, University of Ljubljana, Aškerčeva 7, SI-1000 Ljubljana, Slovenia; 2 Laboratory of Toxicology, Department of Pharmacological Sciences, Università degli Studi di Milano, Via Balzaretti 9, 20133 Milan, Italy

**Keywords:** THP-1, cytokines, IL-8, TNF-α, immunomodulation, NOD1 antagonist, NOD2 antagonist, functional characterization

## Abstract

THP-1 cells express high levels of native functional nucleotide-binding oligomerization domain 1 (NOD1), NOD2, and Toll-like receptor 4 (TLR4) receptors, and have often been used for investigating the immunomodulatory effects of small molecules. We postulated that they would represent an ideal cell-based model for our study, the aim of which was to develop a new in vitro tool for functional characterization of NOD antagonists. NOD antagonists were initially screened for their effect on NOD agonist-induced interleukin-8 (IL-8) release. Next, we examined the extent to which the selected NOD antagonists block the NOD-TLR4 synergistic crosstalk by measuring the effect of NOD antagonism on tumor necrosis factor-α (TNF-α) secretion from doubly activated THP-1 cells. Overall, the results obtained indicate that pro-inflammatory cytokine secretion from THP-1 provides a valuable, simple and reproducible in vitro tool for functional characterization of NOD antagonists.

## 1. Introduction

The cytoplasmic nucleotide-binding oligomerization domain (NOD)-like receptors (NLRs) NOD1 and NOD2 belong to the pattern recognition receptor family and play a vital role in the formation of innate immune response by recognizing distinct pathogen-associated molecular patterns [1,2,3]. The dipeptide d-Glu-*meso*-DAP (iE-DAP) and muramyl dipeptide (MDP) constitute minimal sequences required for activation of NOD1 [4,5,6] and NOD2 [7,8], respectively. Both downstream signaling pathways trigger activation of nuclear factor κB (NF-κB) and mitogen-associated protein kinases, resulting in an inflammatory response [1,2]. iE-DAP and MDP by themselves are rather weak stimulants of immune cells, consequently inducing the release of modest amounts of pro-inflammatory cytokines. However, they were found to synergize with lipopolysaccharide (LPS), a Toll-like receptor 4 (TLR4) agonist, and many other TLR ligands, resulting in increased pro-inflammatory cytokine releasing capacity [9,10,11,12]. NF-κB, which is activated downstream of both TLR and NOD receptors, is a common denominator of both pathways and therefore serves as the primary mediator of synergistic effects of combined stimulation.

**Figure A1:**
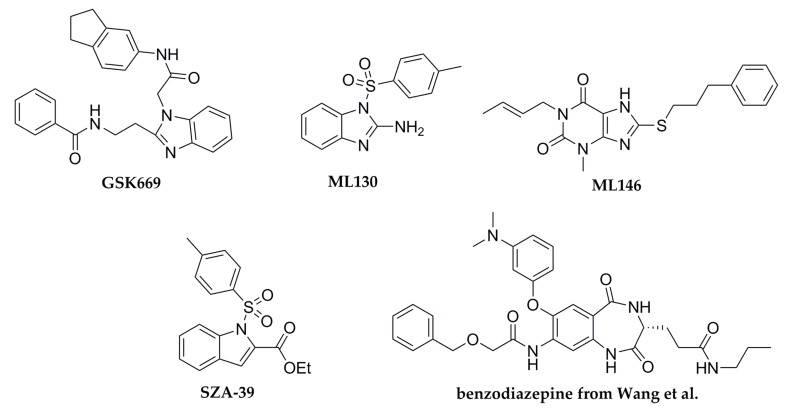
Representative NOD2 antagonist (**GSK669**), NOD1 antagonists (**ML130** and **ML146**), and dual NOD1/NOD2 antagonists (**SZA-39** and benzodiazepine derivative).

Overactivation of NODs has been linked to numerous diseases [13,14,15]. Since antagonism of NOD activity has emerged as a novel approach to treating NOD-related inflammatory and autoimmune diseases [13,14,15], a number of NOD1/NOD2 antagonists have been developed in recent years (depicted in Figure A1). Three diverse structural classes of small molecule NOD1 antagonists have already been reported, namely 2-aminobenzimidazoles [16], purine-2,6-diones [17], and quinazolinones [18]. Of these, Noditinib-1 (ML130), from the 2-aminobenzimidazole class, has been found to selectively inhibit NOD1-dependent NF-κB activation although its mechanism of action has not been elucidated [16,19,20]. In a second screening campaign, compound ML146, belonging to the purine-2,6-diones, was identified as a NOD1 antagonist, exhibiting moderate selectivity [17]. On the other hand, the benzimidazoles represent the only class of currently reported NOD2-selective antagonists [21]. Of note, we and others recently reported the discovery of dual NOD1/NOD2 antagonists (SZA-39) possessing the capacity to inhibit NOD1/2-induced NF-κB activation [22,23].

The NOD1/2 antagonistic activity of compounds and their NOD1 vs. NOD2 selectivity is usually determined using NOD1/2-overexpressing HEK293T cells transfected with a NF-κB-driven luciferase/secreted embryonic alkaline phosphatase (SEAP) reporter gene, usually by pretreating these cells with potential antagonists, subsequent stimulation with NOD1/2 agonists, followed by a simple measurement of luciferase/SEAP activity [13,16,17,22,24]. To extend the analysis beyond reporter gene assays, a few methods have also been developed for assessment of the functional activity of compounds, namely their influence on the authentic downstream effect of NOD-triggered NF-κB activation, the release of interleukin-8 (IL-8). Several cell lines have been considered for functional characterization of NOD ligands. For example, colonic epithelial HCT116 cells express both NOD1 and NOD2 and respond to their activation with increased NF-κB activity, which is reflected in the subsequent secretion of IL-8 [21,25]. Unfortunately, they do not respond to TLR4 agonists and cannot be utilized to investigate how NOD antagonists modulate NOD-TLR synergy. Next, human breast cancer epithelial cell lines MCF-7 overexpressing NOD1 or NOD2 have been used in a similar fashion for characterization of NOD1/2 ligands [26,27]. The major limitations of the MCF-7 assay include the need for overexpression as well as the use of cycloheximide as an IL-8-releasing adjuvant [26,27]. Lastly, in vitro assays using freshly isolated peripheral blood mononuclear cells/monocytes from human blood have also been utilized [28,29,30,31,32]; however, such assays are somewhat inconvenient due to a lengthy isolation procedure, which limits broader application. It is worth noting that assays using primary cells are also highly susceptible to biological donor-to-donor variability.

THP-1 cells derived from an acute monocytic leukemia patient have been used in a variety of studies [33,34] and have been proven to be useful in investigating the immunomodulatory effects of small molecules [35], including NOD agonists [28,36]. In addition to TLR4, THP-1 cells also express high levels of native functional NOD1 and NOD2 and respond to NOD stimulation by IL-8 secretion, due to which they constitute a viable alternative to the aforementioned cell lines [9]. Based on this information, we postulated that THP-1 cells would represent an ideal cell-based model for our present study. Specifically, our aim was to develop a new in vitro assay for functional characterization of validated NOD antagonists by determining how they modulate pro-inflammatory cytokine secretion from activated THP-1 cells. These antagonists were first screened for their ability to inhibit C12-iE-DAP (NOD1 agonist) and/or MDP (NOD2 agonist) induced IL-8 production from THP-1 cells. Given the fact NOD agonists are known to synergistically act with LPS in terms of pro-inflammatory cytokine secretion, the antagonists were further assessed for their ability to inhibit NOD1/NOD2 agonist-induced TNF-α release from LPS-stimulated THP-1 cells. Overall, the results obtained indicated that pro-inflammatory cytokine secretion provides a valuable in vitro tool for functional characterization of NOD antagonists to supplement the results obtained in reporter gene assays.

## 2. Results and Discussion

### 2.1. Effect of Selected Chemicals on Cell Viability

All tested compounds have previously been shown to be well-tolerated by HEK-Blue NOD1 and HEK-Blue NOD2 cells, since they were devoid of cytotoxicity in an 3-(4,5-dimethylthiazol-2-yl)-5-(3-carboxymethoxyphenyl)-2-(4-sulfophenyl)-2H-tetrazolium (MTS) assay [16,17,21,22]. Nevertheless, lactate dehydrogenase (LDH) leakage was initially assessed to evaluate the cytotoxicity of the tested compounds for THP-1 cells (data not shown). Results confirmed that none of the compounds were cytotoxic at the maximum concentration tested (50 µM) with the exception of GSK669 that showed cytotoxicity at concentrations ≥ 10 µM.

### 2.2. Determination of Optimal Experimental Conditions for Screening (Dose-Finding Study)

**Figure 1:**
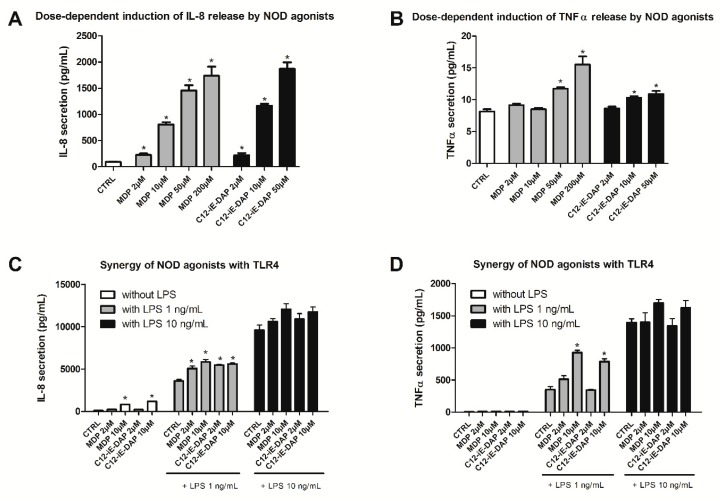
Dose-dependent effect of NOD1/NOD2 agonists on IL-8 (**A**) and TNF-α (**B**) secretion from THP-1 cells. IL-8 and TNF-α release from THP-1 cells were measured following 20 h treatment with increasing concentrations (up to 200 µM) of either C12-iE-DAP (NOD1 agonist) or MDP (NOD2 agonist). Columns represent means of duplicates ± SD of three independent experiments; * *p* < 0.05 vs. CTRL (Mann–Whitney test). Synergy of NOD1/NOD2 agonists and LPS (TLR4 agonist) on IL-8 (**C**) and TNF-α (**D**) secretion. IL-8 and TNF-α release from THP-1 cells were measured following 20 h treatment with either C12-iE-DAP or MDP, both at 2 and 10 µM, in the presence or absence of increasing concentrations of LPS (1 or 10 ng/mL). Columns represent means of duplicates ± SD of three independent experiments; * *p* < 0.05 vs. CTRL (Mann–Whitney test).

THP-1 cells represent a convenient, robust, and reproducible alternative, which enables intra-assay comparison. A consistent protocol is of key importance to evaluate the compounds in a reproducible and unbiased manner. Since cell densities have been shown to affect the outcome of the assay, with the release of cytokines being considerably diminished at low cell densities [37], a cell density of 10^6^ was chosen as optimal, while passaging was performed in agreement with the protocol for the human cell line activation test (h-CLAT) [38]. Recognition of an NOD1/NOD2 agonist triggers a signaling pathway leading to the activation of NF-κB and the production of pro-inflammatory cytokines (e.g., TNF-α, IL-1β, IL-6, IL-8) [9]. To define the optimal experimental conditions, preliminary experiments were conducted with C12-iE-DAP (lauroyl-γ-d-glutamyl-*meso*-diaminopimelic acid), an acylated derivative of iE-DAP, as a reference NOD1 agonist, and MDP as a NOD2 agonist. They were initially screened for their ability to induce IL-8 and TNF-α release from naive THP-1 cells (shown in Figure 1A,B). For isolated stimulation of NOD2 and NOD1 receptors, increasing concentrations of MDP (2–200 µM) and C12-iE-DAP (2–50 µM) were used, and the exposure time of 20 h was chosen based on previous experiments using THP-1 cells [28,39].

Stimulation of cells with C12-iE-DAP/MDP brought about a dose-dependent increase in IL-8 release at all tested concentrations. On the other hand, neither MDP nor C12-iE-DAP produced substantial TNF-α release by themselves, suggesting that this cytokine is not a suitable biomarker for functional evaluation of NOD antagonists. From these preliminary data, the maximum concentration of 10 µM of either NOD1 or NOD2 agonist were chosen for further evaluations of selected NOD antagonists.

Previous work demonstrated that co-stimulation of NODs and TLRs brought about a substantial increase in cytokine production (e.g., IL-8, TNF-α) [36]. To corroborate these results in our assay, we investigated how selected representative NOD1 and NOD2 agonists modulate LPS-induced cytokine secretion from THP-1 cells (shown in Figure 1C,D). THP-1 cells were treated with MDP and C12-iE-DAP (both at 2 µM and 10 µM), alone or in combination with 1 or 10 ng/mL LPS. IL-8 and TNF-α release were assessed 20 h later. As expected, an overwhelming response was observed in terms of IL-8 secretion on co-stimulation with either concentrations of LPS and, therefore, we deemed this cytokine not suitable for determining the effect of NOD antagonists on NOD–TLR4 synergy. On the contrary, in agreement with previous studies, an evident synergistic effect on LPS-induced TNF-α secretion was observed. Stimulation of THP-1 either with MDP or C12-iE-DAP (both at 10 µM) in combination with 1 ng/mL of LPS significantly potentiated the production of TNF-α.

**Figure 2:**
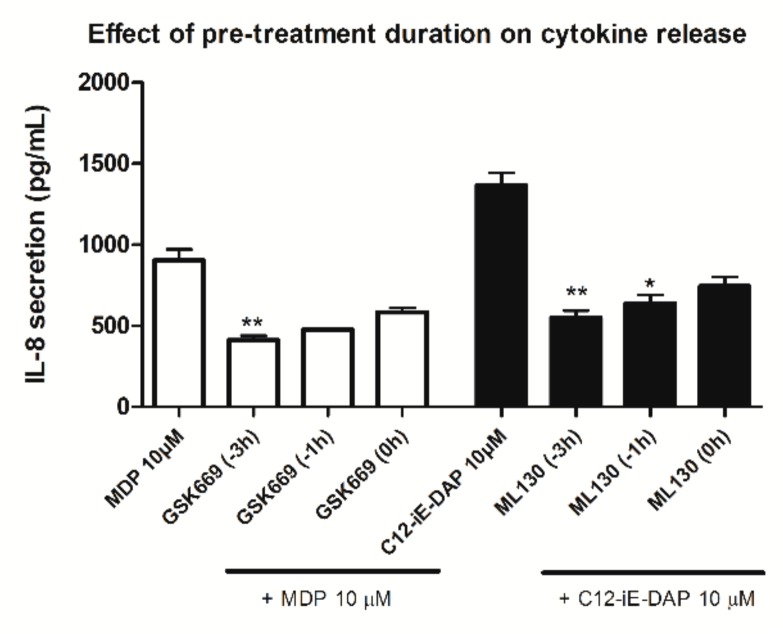
Effect of pretreatment duration with representative NOD2 and NOD1 antagonists on IL-8 secretion from NOD agonist-stimulated THP-1 cells. IL-8 production from THP-1 cells was measured following pretreatment with selected NOD2 or NOD1 antagonists (at 5 µM) for 0, 1, or 3 h before the addition of either MDP or C12-iE-DAP (both at 10 µM), and then incubated for 20 h. Columns represent means of duplicates ± SD of three independent experiments. * *p* < 0.05 and ** *p* < 0.01 vs. MDP/C12-iE-DAP-treated cells (Kruskal–Wallis test and post hoc Dunn’s multiple comparison test).

We addressed the possible effect of NOD antagonist pretreatment duration on the extent of inhibition of cytokine release using the selective bona fide NOD1 and NOD2 antagonists, respectively, ML130 and GSK669. The THP-1 cells were pretreated with 5 µM of ML130 or GSK669 (this concentration was chosen as it corresponds to their respective IC_50_ values) for 0, 1, or 3 h; they were then stimulated with the corresponding NOD agonist (10 µM) and IL-8 release was determined after 20 h (Figure 2). The duration of pretreatment did affect the performance of NOD antagonists, albeit to a minor extent. The maximum inhibition was achieved with pretreatment lasting 3 h, however, 1 h of pretreatment was chosen for reasons of convenience.

### 2.3. Effect of NOD Antagonists on IL-8 Secretion from Stimulated THP-1 Cells

**Figure 3:**
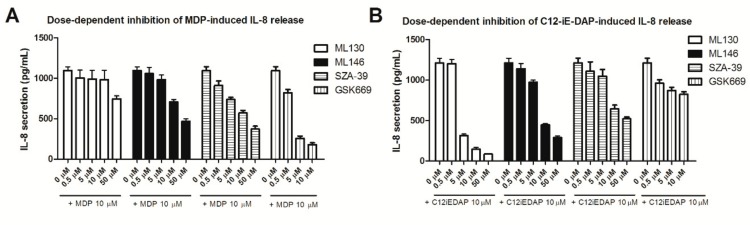
Dose-dependent effect of representative NOD antagonists (ML130, ML146, SZA-39, and GSK669) on (**A**) NOD2 agonist- and (**B**) NOD1 agonist-induced IL-8 secretion from THP-1 cells. IL-8 release from THP-1 cells was measured following pretreatment with selected NOD antagonists at increasing concentrations for 1 h before the addition of either MDP or C12-iE-DAP (both at 10 µM) and then incubated for 20 h. Columns represent means of duplicates ± SD of three independent experiments.

Treatment of THP-1 cells with the selected NOD antagonists alone did not produce substantial IL-8 release and only negligible TNF-α secretion at the maximum concentration tested (data not shown). Having established that the compounds themselves do not affect the cytokine secretion, we investigated the dose-dependent effect of selected NOD1, NOD2, and dual NOD1/2 antagonists on NOD agonist-induced IL-8 release. Pretreatment of THP-1 with increasing concentrations of NOD antagonists (0.5–50 µM) resulted in dose-dependent suppression of IL-8 release from NOD agonist-stimulated THP-1 cells (Figure 3A,B).

**Figure A2:**
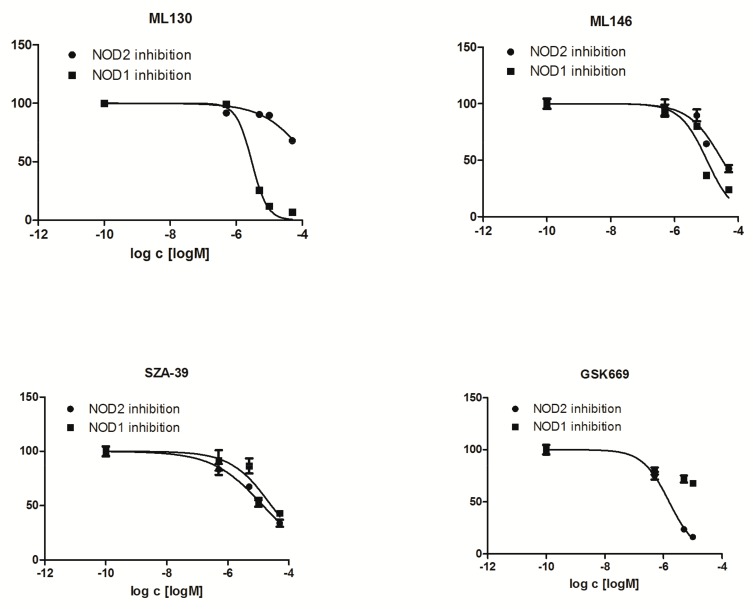
Inhibition curves of investigated NOD antagonists representing their effect on IL-8 secretion from NOD agonist-activated THP-1.

Notably, GSK669 was not tested at the highest concentration (50 µM) due to cytotoxicity. As expected, the results demonstrated predominant actions of ML130 and ML146 towards NOD1 agonist-induced cytokine release, while GSK669 mostly affected NOD2 agonist-induced release. The dual antagonist SZA-39 antagonized the response to both stimuli. Overall, all tested compounds induced a dose-dependent inhibitory effect on IL-8 release, following a nonlinear semilogarithmic model (Figure A2); their IC_50_ values were also determined. The relationship between IC_50_ values determined in previous in vitro reporter assays using commercially available NOD-overexpressing HEK cell lines and the IC_50_ values obtained in our functional assay are summarized in Table 1 and shown for comparison. The obtained results in THP-1 cells revealed a low micromolar IC_50_ of the reference NOD1 antagonist ML130 on NOD1 (IC_50_ = 2.97 ± 0.31 µM) and selectivity towards NOD2 (IC_50_ > 50 µM), which is in rather good agreement with the previously reported activities in reporter gene assays [22]. Similarly, the results for ML146 (IC_50_ (NOD1) = 10.5 ± 1.32 µM; IC_50_ (NOD2) = 32.2 ± 5.16 µM) indicated a somewhat lower activity as opposed to those obtained in reporter gene assays [22]. By contrast, GSK669 possesses a low micromolar IC_50_ on NOD2 (IC_50_ = 1.57 ± 0.15 µM) and is selective towards NOD1 (IC_50_ > 50 µM), thus representing a good match to the activities measured in HEK reporter cell lines [21]. Finally, the dual NOD1/NOD2 antagonist SZA-39 showed a slightly weaker activity in THP-1 cells (IC_50_ (NOD1) = 27.5 ± 6.85 µM; IC_50_ (NOD2) = 14.4 ± 1.97 µM), compared to those measured in HEK cells [22].

### 2.4. Effect of Selected NOD1/NOD2 Antagonists on NOD1/2-TLR4 Synergy

**Figure 4:**
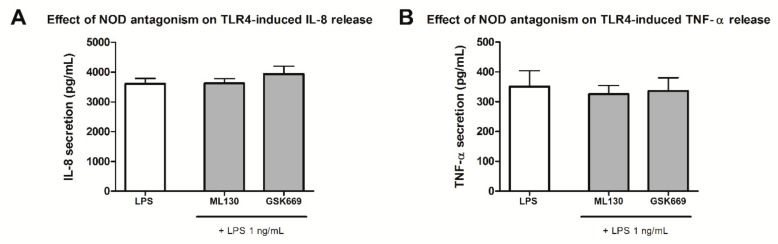
Effect of NOD antagonism on TLR4-induced pro-inflammatory cytokine release. IL-8 (**A**) and TNF-α (**B**) release from THP-1 cells were measured following pretreatment with selected NOD antagonists, ML130 and GSK669 (both at 10 µM), for 1 h before the addition of TLR4 agonist LPS (1 ng/mL) and then incubated for 20 h. Columns represent means of duplicates ± SD of three independent experiments. *p* > 0.05 vs. LPS-treated cells (Kruskal–Wallis test and post hoc Dunn’s multiple comparison test).

Combined NOD/TLR stimulation reflects the scenario of pathological conditions of infection and chronic inflammation, thus recapitulating the innate immune responses to invading bacteria. Previous work has shown that co-stimulation of NOD1/2 and TLR4 brought about a substantial increase in pro-inflammatory cytokine production (e.g., IL-8, TNF-α) [40]. This prompted us to investigate whether our assay has the capacity to elucidate, and if so, to what extent the NOD antagonists block such a synergistic response. Specifically, we determined the effect of NOD antagonism on TNF-α secretion from activated THP-1 cells. Firstly, in order to exclude a possible effect of NOD antagonists on the TLR4 pathway and ascertain the compounds’ selectivity profile, an assay measuring the effect of NOD1/NOD2 antagonists on TLR4-dependent IL-8 and TNF-α release was utilized. Evidently, the pretreatment with NOD antagonists did not prevent the LPS-elicited pro-inflammatory cytokine release from THP-1 (Figure 4).

**Figure 5:**
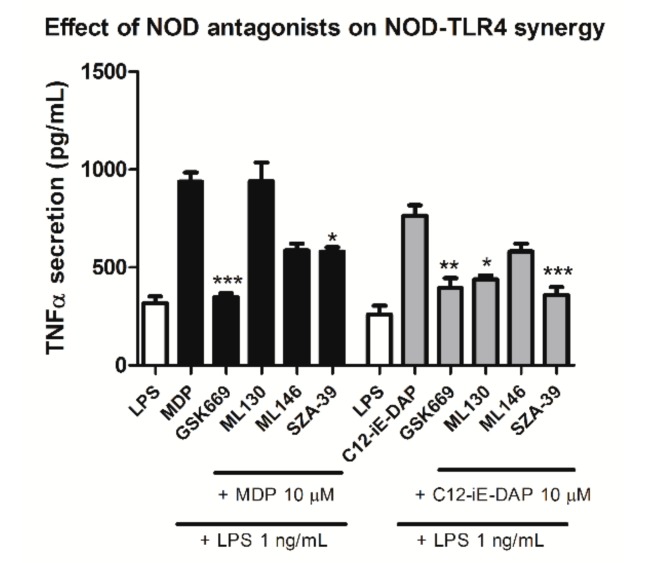
Effect of selected NOD1/NOD2 antagonists on NOD1/2–TLR4 synergy. The THP-1 cells were preincubated for 1 h with NOD antagonists (at 10 µM) before being stimulated with either C12-iE-DAP or MDP (both at 10 µM) and 1 ng/mL LPS for 20 h, followed by determination of TNF-α release in the supernatant. Columns represent means of duplicates ± SD of three independent experiments. * *p* < 0.05, ** *p* < 0.01 and *** *p* < 0.001 vs. MDP/LPS- or C12-iE-DAP/LPS-treated cells (Kruskal-Wallis test and post hoc Dunn’s multiple comparison test).

Secondly, the observed diminished levels of TNF-α production triggered by combined stimulation of THP-1 cells clearly demonstrates that antagonists of the NOD1/2 signaling pathway successfully suppressed NOD–TLR4 synergy (Figure 5). Namely, in co-stimulated cells, GSK669 reduced TNF-α release to the level induced by the TLR4 agonist alone. In accordance, the synergistic effect induced by C12-iE-DAP and LPS was markedly suppressed in NOD1-antagonized cells. These results corroborate those obtained by Uehara et al., though it should be noted they employed RNA interference technology to suppress NOD1/NOD2 [36]. Importantly, our findings indicate that NOD–TLR4 crosstalk can be completely abolished by small molecules.

## 3. Materials and Methods

### 3.1. Test Chemicals

C12-iE-DAP (a synthetic NOD1 agonist) and MDP (NOD2 agonist) were obtained from InvivoGen, Inc., (San Diego, CA, USA); LPS (from *Escherichia coli* serotype 0127:B8) was from Sigma-Aldrich, Inc. (St. Louis, MO, USA). NOD antagonists were selected based on the information provided by published studies. The NOD1, NOD2, or dual NOD1/NOD2 antagonistic properties of these compounds were previously identified using the established commercially available HEK-Blue NOD cell lines [21,22]. The NOD1 antagonist, ML-146, was purchased from Enamine Ltd. (Kyiv, Ukraine). The NOD1 antagonist, Noditinib-1 (ML-130), was synthesized as described [19]. The NOD2 antagonist, GSK669, was synthesized as described [21]. The NOD1/2 dual antagonist, SZA-39, was synthesized as described [22]. The HPLC purity of all pharmacologically investigated compounds was >95%. Stock solutions of chemicals were prepared in DMSO before use and the final concentration of DMSO never exceeded 0.2%.

### 3.2. Cell Culture

For all experiments, THP-1 cells (Istituto Zooprofilattico di Brescia, Brescia, Italy) were diluted to 10^6^ cells/mL in RPMI 1640 containing 2 mM L-glutamine, 0.1 mg/mL streptomycin, 100 IU/mL penicillin, 50 μM 2-mercaptoethanol, and supplemented with 10% heat-inactivated fetal calf serum (media) and cultured at 37 °C in 5% CO_2_. The experiments were carried out on passages 3–12. To evaluate IL-8 and TNF-α production, cultures were set up in 24-well culture plates containing 500 μL of cells. They were pretreated with selected NOD antagonists at increasing concentrations for 1 h before the addition of either MDP or C12-iE-DAP (both at 10 µM) and then incubated for 20 h. Each concentration was tested in duplicate, and untreated cells were exposed to DMSO, which represented the vehicle control (not exceeding 0.2% final concentration). In synergy studies, cells were additionally stimulated with lipopolysaccharide (LPS, from *Escherichia coli* serotype 0127:B8, Sigma) at a final concentration of 1–10 ng/mL, as indicated in the figure legends. Cell-free supernatants were collected at 20 h by centrifugation at 3000 rpm for 5 min and stored at −80 °C.

### 3.3. Cell Viability

Prior to investigating the effects of selected NOD antagonists on cytokine production, their potential cytotoxicity for THP-1 cells was assessed. Cell viability was assessed by leakage of LDH from damaged cells. LDH is a well-known indicator of cell membrane integrity and cell viability [41]. Cells were treated for 20 h with the compound of interest at concentrations of up to 50 µM. LDH activity was determined in cell-free supernatants using a commercially available colorimetric kit (Takara Bio Inc., Kusatsu, Japan). Results are expressed as OD.

### 3.4. Cytokine Production (ELISA)

IL-8 and TNF-α release from THP-1 cells were measured in cell-free supernatants obtained by centrifugation at 3000 rpm for 5 min and stored at −80 °C until measurement. IL-8 and TNF-α production were assessed by specific sandwich ELISA (Immunotools, Friesoythe, Germany; eBioScience/R&D Systems, Minneapolis, MN, USA). Results are expressed in pg/mL. The limit of detection was 15.6 pg/mL for IL-8 and 4 pg/mL for TNF-α.

### 3.5. Data Analysis and Statistics

All experiments were performed at least three times, with average values expressed as means ± standard deviation (SD). Statistical analyses were performed using GraphPad Prism 6 (La Jolla, CA, USA). Statistical significance was determined either with the Mann-Whitney or Kruskal-Wallis followed by a post hoc Dunn’s multiple comparison test, as indicated in the figure legends. Differences were considered nonsignificant for *p* > 0.05, significant (*) for *p* < 0.05, very significant (**) for *p* < 0.01 and extremely significant (***) for *p* < 0.001. IC_50_ values of NOD1/2 inhibition were calculated by a nonlinear regression model using GraphPad Prism 6 software.

## 4. Conclusions

In this study, we clearly demonstrated that the THP-1 assay, as described above, represents a convenient screening tool for functional activity of NOD antagonists. While IL-8 release proved to be a suitable biomarker for functional characterization of NOD antagonism, the TNF-α release additionally provides an estimate of the NOD antagonists’ capacity to block NOD-TLR crosstalk. Overall, the results obtained indicate that the pro-inflammatory cytokine secretion profile from THP-1 cells constitutes a valuable, simple, and reproducible in vitro tool for functional characterization of NOD antagonists to supplement the results of reporter gene assays.

## Figures and Tables

**Table 1:** NOD1/NOD2 antagonistic activities by selected compounds as reported in the literature.

Cpd	HEK-NOD1 IC_50_ [μM]	HEK-NOD2 IC_50_ [μM]
ML130	0.771 [22]	54.9 [22]
ML146	4.35 [22]	12.9 [22]
GSK669	>50 [21]	3.2 [21]
SZA-39	5.74 [22]	6.45 [22]

## Abbreviations

C12-iE-DAP	lauroyl-γ-d-glutamyl-*meso*-diaminopimelic acid
h-CLAT	human cell line activation test
iE-DAP	γ-d-glutamyl-*meso*-diaminopimelic acid
IL	interleukin
LDH	lactate dehydrogenase
LPS	lipopolysaccharide
MDP	muramyl dipeptide
MTS	3-(4,5-dimethylthiazol-2-yl)-5-(3-carboxymethoxyphenyl)-2-(4-sulfophenyl)-2H-tetrazolium
NF-κB	nuclear factor κB
NLR	NOD-like receptor
NOD	nucleotide-binding oligomerization domain
PRR	pattern recognition receptors
SEAP	secreted embryonic alkaline phosphatase
TLR	toll-like receptor
TNF-α	tumor necrosis factor-α
